# Comparative Analysis of Heat-Tolerant and Heat-Susceptible Rice Highlights the Role of *OsNCED1* Gene in Heat Stress Tolerance

**DOI:** 10.3390/plants11081062

**Published:** 2022-04-13

**Authors:** Huang Zhou, Yingfeng Wang, Yijin Zhang, Yunhua Xiao, Xiong Liu, Huabing Deng, Xuedan Lu, Wenbang Tang, Guilian Zhang

**Affiliations:** 1 College of Agronomy, Hunan Agricultural University, Changsha 410128, China; zhouhuangbaba@163.com (H.Z.); wangyingfeng229@163.com (Y.W.); z1762233735@163.com (Y.Z.); xyhkemy@163.com (Y.X.); xiongliu@whu.edu.cn (X.L.); denghuabing@126.com (H.D.); luxuedan1@126.com (X.L.); 2 Hunan Provincial Key Laboratory of Rice and Rapeseed Breeding for Disease Resistance, Hunan Agricultural University, Changsha 410128, China; 3 State Key Laboratory of Hybrid Rice, Hunan Hybrid Rice Research Centre, Changsha 410125, China

**Keywords:** rice, heading and flowering stage, heat stress, transcriptome, *OsNCED1*, antioxidant enzyme

## Abstract

To elucidate the mechanism underlying the response of rice to heat stress (HS), the transcriptome profile of panicles was comparatively analyzed between the heat-tolerant line 252 (HTL252) and heat-susceptible line 082 (HSL082), two rice recombinant inbred lines (RILs). Our differentially expressed gene (DEG) analysis revealed that the DEGs are mainly associated with protein binding, catalysis, stress response, and cellular process. The MapMan analysis demonstrated that the heat-responsive (HR) genes for heat shock proteins, transcription factors, development, and phytohormones are specifically induced in HTL252 under HS. Based on the DEG analysis, the key gene *OsNCED1* (*Os02g0704000*), which was induced under HS, was selected for further functional validation. Moreover, 9-cis-epoxycarotenoid dioxygenase (NCED) is a key rate-limiting enzyme in the ABA biosynthetic pathway. Overexpression of *OsNCED1* improved the HS tolerance of rice at the heading and flowering stage. *OsNCED1*-overexpression plants exhibited significant increases in pollen viability, seed setting rate, superoxide dismutase (SOD) and peroxidase (POD) activities, while significantly lower electrolyte leakage and malondialdehyde (MDA) content relative to the wild type (WT). These results suggested that *OsNCED1* overexpression can improve the heat tolerance of rice by enhancing the antioxidant capacity. Overall, this study lays a foundation for revealing the molecular regulatory mechanism underlying the response of rice to prolonged HS.

## 1. Introduction

Heat stress (HS) resulting from high temperature is an increasingly serious problem in global agriculture that greatly restricts the growth and productivity of plants. HS can cause a series of changes in plants, such as prolonged seed filling, reduction in cell volume, closure of stomata, changes in water and osmotic substances, oxidative stress, and protein denaturation, which can negatively influence the growth and development of plants and drastically reduce the yield and quality of crops [1,2,3]. It has been reported that the increase of 1 °C in temperature will decrease the yield of wheat, corn, and rice by 6%, 7.4%, and 3.2%, respectively [4]. Rice (*Oryza sativa* L.) is an important crop providing staple food for more than half of the world’s population. China is the largest country of rice production and consumption. Due to the influence of subtropical heat, persistent extremely high temperature frequently occurs in the middle and lower reaches of the Yangtze River (the main rice production area in China) at the heading and grain filling stages (mid-July to mid-late August), thus causing serious yield reduction or even total crop failure [5,6].

Clarification of the molecular mechanism underlying the response of plants to HS will greatly help to improve the tolerance of plants to environmental stress through genetic manipulation. There has been genomic and genetic evidence suggesting that many proteins are involved in plant HS response [7,8]. In rice, numerous HS responsive genes have been identified, including heat shock proteins (HSPs), heat shock transcription factors (Hsfs), antioxidant enzymes, stress transcription factors (TFs), and other proteins induced under stress conditions [9,10,11]. These genes can protect cells from damage through their production of some important metabolic proteins, and they also play important roles in gene regulation and signaling. Most research on the changes in gene expression induced by HS in rice was conducted for only a short period of time (generally 1–2 h) [8,12,13,14,15]. However, the actual high temperature usually lasts for a long time in real production, and little is known about the response of these genes under prolonged HS.

In this study, to explore the molecular basis for rice response to prolonged HS, two extreme recombinant inbred lines (RILs), the heat-tolerant line 252 (HTL252) and heat-susceptible line 082 (HSL082), were selected to comparatively analyze the differences in gene expression after 5 days of HS at the heading and flowering stage by transcriptomic approaches. In addition, based on the analysis of differentially expressed genes (DEGs), the *OsNCED1* (*Os02g0704000*) gene induced under HS was selected for further functional validation. Our results may provide a valuable contribution to the understanding of the molecular mechanism of rice response to prolonged HS and, therefore, to the genetic improvement of HS tolerance in crops.

## 2. Materials and Methods

### 2.1. Plant Materials and Heat-Stress Treatment

The heat-tolerant line 252 (HTL252) and heat-susceptible line 082 (HSL082) were two extreme individuals of the recombinant inbred lines (RILs) derived from a cross between the heat-tolerant rice line 996 and the sensitive line 4628; the heat-tolerant line 996 was reported highly heat-tolerant, and it exhibited better anther dehiscence and pollen fertility rate than heat-sensitive line 4628 under HS. HTL252, HSL082, Nipponbare (WT), and two *OsNCED1* overexpression lines (OE-1 and OE-2) were cultivated in plastic pots placed in a ventilated greenhouse. On the first day of heading, the pots were moved to a controlled growth chamber with HS treatment (37 °C day/30 °C night cycle) for 5 d. The light intensity was 200 μmol /(m^−2^·s^−1^); the light period was 12 h each day, and the relative humidity was 65–75% throughout the experimental period. The control pots were placed in a separate controlled environment chamber with control temperature (CT 30 °C day/25 °C night cycle) and otherwise identical conditions for the same experimental period. The greenhouse temperature during growth (before treatment) was the same as that of the control pots. HTL252 and HSL082 were grown in 6 pots with three plants for each pot (3 pots for HS treatment and 3 pots for CT treatment), while WT, OE-1, and OE-2 were grown in 8 pots with 3 plants for each pot (4 pots for HS treatment and 4 pots for CT treatment).

### 2.2. RNA Isolation, Microarray Hybridization, Signal Scanning, and Normalization

After 5 d of continuous treatment, young panicles were collected from HTL252 and HSL082, immediately frozen in liquid nitrogen, and then stored at −80 °C before RNA isolation. Total RNA was isolated with Trizol (Invitrogen, Carlsbad, CA, USA) and then purified with the QIAGEN RNeasy kit (Qiagen, lnc, Valencia, CA, USA) according to the manufacturer’s instructions. Array hybridization and wash were conducted by using GeneChip^®^ Hybridization, Wash and Stain Kit in Hybridization Oven 645 and Fluidics Station 450 according to the manufacturer’s instructions. Scanning of the slides was performed by GeneChip^®^ Scanner 3000 and Command Console Software 4.0 with default parameters. MAS 5.0 algorithm, Gene Spring Software 11.0 (Agilent technologies, Santa Clara, CA, USA), was used to normalize the raw data.

### 2.3. Data Filtering, Clustering and Functional Classification

The DEGs with four or more folds of changes were identified. The Venn diagram analysis and hierarchical clustering (HCL) were performed to examine the expression pattern of genes with significant changes during heat treatment. The genes were classified as previously described, based on gene ontology [16]. The pathway was analyzed with MapMan [17]. Further identification of the functionally characterized genes was conducted by using the OGRO database (http://qtaro.abr.affrc.go.jp/ogro, 5 April 2016).

### 2.4. Quantitative Real-Time PCR

Total RNA was extracted, and the cDNA was synthesized with the previously described methods. Primer sequences were designed for amplicons of each gene by using Primer Premier 6.0 (Supplementary Table S1). Rice 18S gene was used as the internal control. Then qRT-PCR was carried out by following the methods reported by Rerksiri et al. [18]. Three technical replicates and two biological replicates were performed at each sampling for each gene. The gene expression was quantified by using the 2^−^^ΔΔCT^ method [19], and the data were compared with those of the internal control.

### 2.5. Cloning of OsNCED1 CDS

The total RNA of Nipponbare was extracted and reverse transcribed into cDNA, and then PCR amplification was carried out with cDNA as the template. The amplification procedure consisted of pre-denaturation at 98 °C for 5 min, followed by denaturation at 98 °C for 30 s, annealing at 58 °C for 30 s, extension at 72 °C for 2 min, a total of 34 cycles, and then final extension at 72 °C for 7 min and preservation at 16°C. After electrophoretic determination of the PCR products, the target fragment was recovered, then connected to pMD19-T cloning vector, and transformed into *E. coli* DH5α. After PCR detection, the positive clones were sequenced, and the bacteria with correct sequencing were frozen in glycerol.

### 2.6. Generation of OsNCED1 Overexpression Lines

The target CDS fragment of *OsNCED1* was cloned into a pCAMBIA1300 plant overexpression vector carrying a maize ubiquitin promoter, resulting in the generation of a plasmid named pCAMBIA1300-OsNCED1. Double digestion was performed with KPN I; and BamH I; for vector construction. The resulting plasmid was introduced in *Agrobacterium* tumefaciens strain EHA105, which was used for the genetic transformation of plants from the rice variety Nipponbare.

Positive transgenic plants were screened and further identified through PCR and qPCR, and 10 homozygous lines were obtained, which were designated as OE-1 to OE-10. Two independent lines (OE-1 and OE-2), which had particularly high transcription levels of *OsNCED1*, were selected to be further analyzed.

### 2.7. Determination of Expression Pattern and Transcription Levels of OsNCED1 and ABA Signaling Related Genes

The young panicles, stems, leaves, and leaf sheaths of HTL252 and HSL082 were collected on the 5th day of HS treatment to determine the expression pattern of *OsNCED1* in three biological replicates. The leaves of WT, OE-1, and OE-2 treated with control and HS for 5 d were used to determine the transcription levels of *OsNCED1* and ABA signaling related genes, using qRT-PCR, in three biological replicates. Total RNA from these samples was extracted, and cDNA was synthesized as previously described. The cDNA of different treatments was detected by qRT-PCR with the ubiquitin gene as an internal reference. The qRT-PCR was performed according to the methods previously reported by Rerksiri et al. [18]. The detection was repeated for 3 times to determine the expression pattern of the *OsNCED1* gene under HS.

### 2.8. Determination of Pollen Viability, Seed Setting Rate, Cell Membrane Damage, and Oxidative Stress in Transgenic Rice Plants under HS

Spikelets from Nipponbare (WT), OE-1, and OE-2 were collected to determine the pollen viability and seed setting rate, and leaves were used to determine the MDA content, electrolyte leakage, and POD and SOD activities.

The pollen viability was measured on the third day after HS treatment, and 5 open spikelets on that day were selected. The pollen from the anthers of these spikelets was squeezed and mixed on the slide, which was then stained with 1% I_2_-KI and placed under a microscope to observe three visual fields, and the average ratio (%) of stained pollen grains to total pollen grains in three visual fields was calculated to indicate pollen vitality.

The seed setting rate was investigated at maturity stage, when all grains had brown shells [20]. All marked panicles were collected, and the total number of grains and the number of filled grains were investigated, and the average seed setting rate of marked panicles was calculated. The grains were pressed with thumb and forefinger or checked by opening the lemma and palea to determine filled grains and empty grains.

Malondialdehyde (MDA) is a product of membrane lipid peroxidation and a marker of cell membrane damage. Electrolyte leakage is an effective index to evaluate cell membrane permeability. The electrolyte leakage, MDA content, and activities of major antioxidant enzymes were determined at 1, 3, and 5 days of HS treatment. The SOD and POD activities were measured according to Zhang et al. [21]. One unit of SOD activity was defined as the amount of enzyme per enzyme extract sample that caused 50% inhibition of the reduction of NBT. The POD activity was calculated with the change of OD_470_ per minute of 0.01 as a relative enzyme activity unit. The electrolyte leakage and MDA content were measured according to the previously described procedure [22]. The electrolyte leakage was calculated with the following formula: relative conductivity (%) = (treatment conductivity − control conductivity)/(boiling conductivity − control conductivity) × 100%. All experiments were conducted in three biological replicates.

### 2.9. Statistical Analysis

Data collation was performed with Microsoft Excel (Microsoft Corporation, Redmond, WA, USA). Student’s *t*-test and analysis of variance (ANOVA) were conducted by using SPSS (version 17.0, SPSS Inc., Chicago, IL, USA). Graphs were constructed with GraphPad Prism (v 8.0.2 GraphPad Software Inc., San Diego, CA, USA).

## 3. Results

### 3.1. Differentially Expressed Gene Analysis under HS

**Figure 1:**
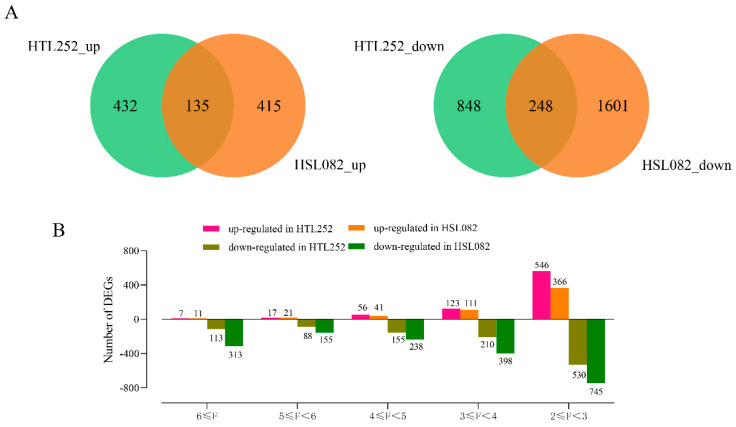
(**A**) Venn diagram of DEGs in HTL252 and HSL082 under HS. HTL252_up indicates the upregulated genes in HTL252, HSL082_up represents the upregulated genes in HSL082, HTL252_down indicates the downregulated genes in HTL252, and HSL082_down stands for the downregulated genes in HSL082. (**B**) Distribution of DEGs according to fold-changes F. Negative log base 2 changes in fold indicate repression, while positive values represent induction. *Y*-axis indicates the number of upregulated or downregulated DEGs. The part of *Y*-axis above 0 stands for upregulation, and that below 0 represents downregulation. *X*-axis indicates the range of variation of F.

The differentially expressed genes (DEGs) of panicles in rice after 5 days of HS treatment were considered as heat-responsive (HR) genes when they showed over 4-fold changes in expression relative to the control. A total of 1663 and 2399 probe sets exhibited at least four-fold changes in expression (*p* < 0.01) for HTL252 and HSL082, respectively. Among the DEGs in HTL252, 522 genes (567 probe sets) were upregulated and 1016 genes (1096 probe sets) were downregulated (Figure 1A). In contrast, fewer genes were HS-responsive in HSL082, among which 496 genes (550 probe sets) were upregulated and 1707 genes (1849 probe sets) were downregulated (Figure 1A). There were 129 common upregulated genes (135 probe sets) and 241 common downregulated genes (248 probe sets) in both HTL252 and HSL082 (Figure 1A). The two lines showed a different distribution of DEGs based on fold changes in their expression (Figure 1B). For example, although HSL082 had more DEGs with log base 2-fold changes than HTL252, it had fewer highly upregulated genes than HTL252 (Figure 1B).

**Figure 2:**
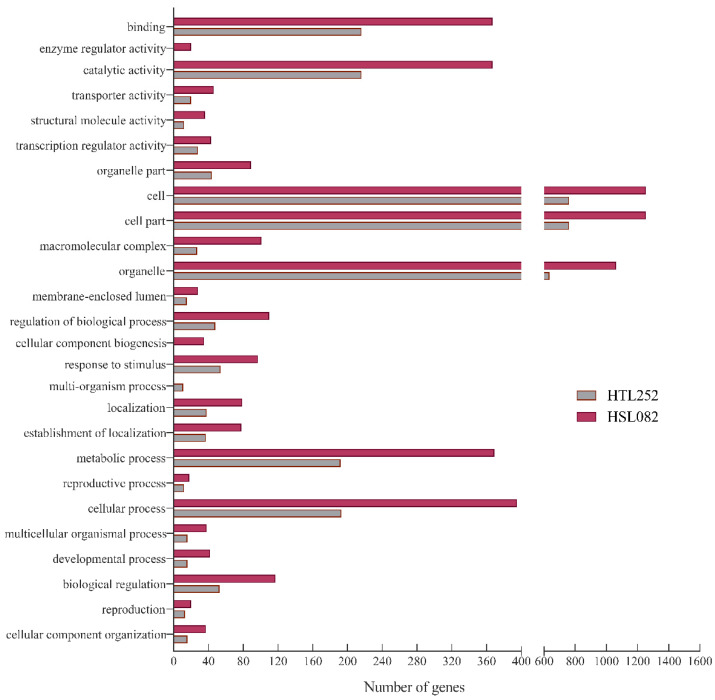
Gene Ontology (GO) classification of 1168 DGEs in HTL252 and 1833 DEGs in HSL082 after 5 days of HS treatment. *Y*-axis shows the GO terms. *X*-axis shows the number of genes enriched to this term.

Functional classification of the 1168 DEGs identified in HTL252 and 1833 DEGs in HSL082 (Figure 2) revealed that these DEGs are mainly associated with cellular process, protein binding, catalysis, and stress response (Figure 2). The MapMan analysis revealed that HS induced the HR genes for TFs, HSPs, development, and phytohormones (Supplementary Tables S2–S4 and S6).

### 3.2. Comparative Analysis between HTL252 and HSL082

In total, 132 and 215 DEGs coding for TFs were respectively characterized in HTL252 and HSL082, which mainly belong to some super protein families, such as AP2/EREBP, basic Helix-Loop-Helix (bHLH), C2C2, C2H2, HB, NAC, MYB, WRKY, and bZIP (Table S2). Most C2C2 genes were upregulated under HS treatment in both lines, among which *Os02g0606200* (*OsBBX4*) and *Os01g0263900* showed much higher expression levels in HTL252 than in HSL082. Significant differences were observed in the expression of MYB-related genes between the two lines. HS treatment upregulated 14 out of the 19 MYB-related genes in HTL252, while downregulated most of the MYB-related genes in HSL082. In addition, most NAC and bZIP genes were upregulated in HTL252 under HS treatment, but they were downregulated in HSL082 (Supplementary Table S2).

**Figure 3:**
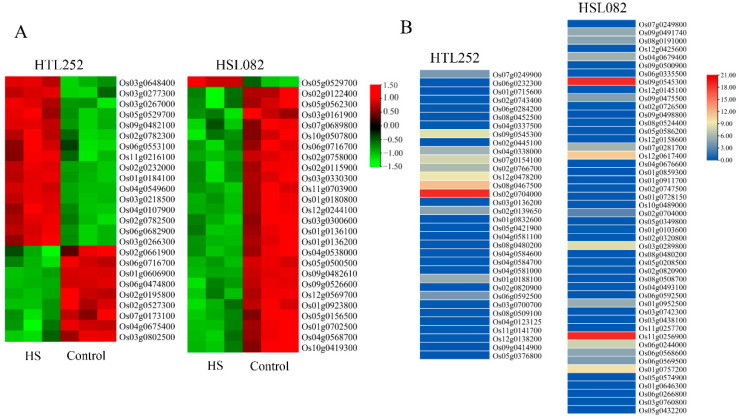
(**A**) Heat map of DEGs associated with heat shock protein genes in HTL252 and HSL082 after 5 d of HS treatment based on the expression levels. The red color represents upregulated genes, and the green color represents downregulated genes. (**B**) Expression profiles of phytohormone-related HR genes based on fold changes. The blue color represents low-level expression, light yellow color represents medium level, and red color represents the highest level. The fold change is the ratio of the signal value of the high temperature sample to that of the optimum temperature sample.

The HR genes involved in abiotic stress response mainly comprise HSPs, DnaJ, and Hsf protein families. HS treatment upregulated 16 of 25 HSPs genes in HTL252, particularly *Os02g0232000* (*OsHsfC2a*), *Os04g0107900* (*OsHSP1*), *Os03g0218500*, *Os02g0782500*, and *Os03g0277300*; meanwhile, it only upregulated one (*Os05g0529700*) gene and downregulated the other 25 HSPs genes in HSL082 (Figure 3A and Suppleemntary Table S3).

The data revealed that the HR genes related to development mainly encode storage proteins, late embryogenesis abundant proteins, and unspecified proteins (Supplementary Table S4). The genes for storage proteins were downregulated by HS treatment in both lines. There were significant differences in the expression of late embryogenesis abundant protein genes between the two lines, which were upregulated in HTL252 but downregulated in HSL082 under HS treatment (Supplementary Table S4). Moreover, the genes related to the HR ubiquitin–proteasome system (UPS) mainly comprised ubiquitin, E2, and RING finger, SCF, and BTB/POZ from the E3 super-family and exhibited much higher expression levels in HTL252 than in HSL082 (Supplementary Table S5).

Phytohormones are critical to the adaptation of plants to various stresses. Our data showed that the phytohormone-related HR genes were mainly involved in the metabolism and regulation of auxin, ABA, gibberelin, salicylic acid (SA), cytokinin (CK), and ethylene (Figure 3B and Supplementary Table S6). Most of the auxin, SA, gibberellin, and ethylene-related genes were downregulated under HS in both lines. In contrast, the expression levels of ABA-related genes were significantly different in the studied lines, since, in HTL252, these genes were upregulated, particularly *Os07g0154100* (*OsNCED4*), *Os02g0766700* (*OsbZIP23*), *Os12g0478200*, *Os08g0467500*, and *Os02g0704000* (*OsNCED1*), while most of them were obviously downregulated in HSL082 (Figure 3B and Supplementary Table S6). These results demonstrated that ABA plays an important role in the response of rice to prolonged HS.

### 3.3. Quantitative Real-Time PCR (qRT-PCR) Verification

**Figure 4:**
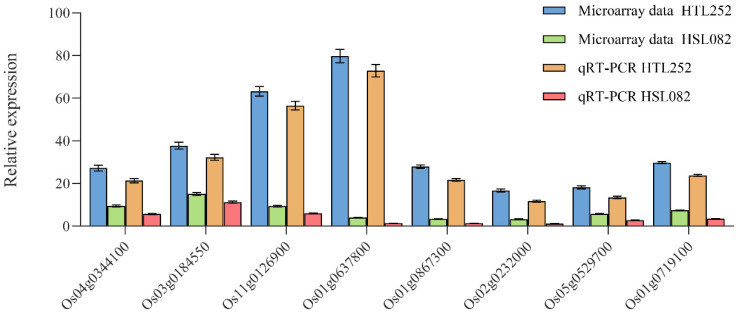
Verification through qRT-PCR. Eight genes were selected for qRT-PCR and compared with the microarray data. For each qRT-PCR, the transcription level of the rice 18S gene in different samples was also assessed. Quantification of gene expression was performed by using the relative quantification method (2^−ΔΔCT^). “Microarray data HTL252” and “Microarray data HSL082” denote the results of HTL252 and HSL082 in the microarray, respectively; “qRT-PCR HTL252” and “qRT-PCR HSL082” represent the qRT-PCR results of HTL252 and HSL082, respectively. Values are mean average ± SE of three replicates.

For validation of the microarray data, eight genes associated with TFs, UPS, HSP, and secondary metabolism were selected to perform quantitative real-time PCR (qRT-PCR). As a result, the qRT-PCR results exhibited good consistency with the microarray data (Figure 4).

### 3.4. Expression Pattern of OsNCED1 Gene under HS

**Figure 5:**
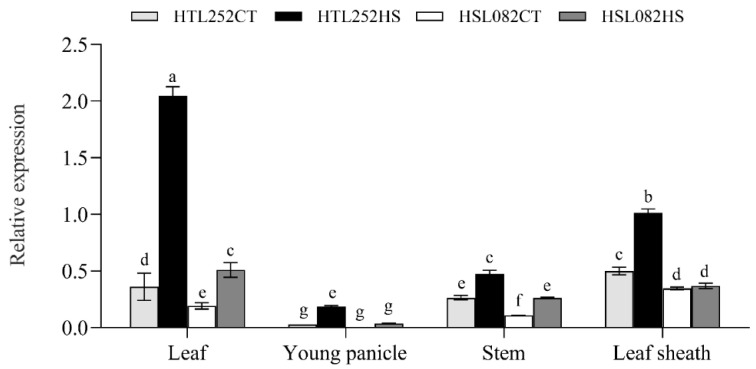
Expression pattern of the *OsNECD1* gene in different tissues of rice plants after 5 d of HS treatment (37 °C day/30 °C night). HTL252CT: HTL252 under control temperature (30 °C day/25 °C night) treatment. HTL252HS: HTL252 after 5 d of HS (37 °C day/30 °C night) treatment. HSL082CT: HSL082 under control temperature (30 °C day/25 °C night) treatment. HSL082HS: HSL082 after 5 d of HS (37 °C day/30 °C night) treatment. Data shown are means ± SE of three independent experiments. Statistical analysis was performed by ANOVA test (*p* < 0.05), and significant differences are indicated by different letters.

Notably, the ABA-related gene *OsNCED1* (*Os02g0704000*) was upregulated under HS in both lines, but its expression was much higher in HTL252 than in HSL082 (Figure 3B). We therefore suspected that its differential expression may be an important reason for the different response of rice to prolonged HS. Compared with the control temperature (CT), HS induced the expression of *OsNCED1* in both lines (Figure 5). On the fifth day of heat treatment, the expression level of *OsNCED1* followed the order of leaf > leaf sheath > stem > young panicle (Figure 5).

### 3.5. Analysis of Pollen Viability and Seed Setting Rate in OsNCED1 Transgenic Rice Plants

**Figure 6:**
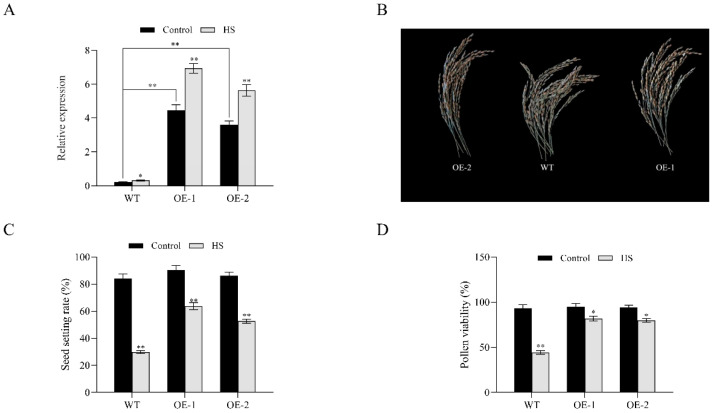
Relative expression of *OsNCED1* in OE-1, OE-2, and WT under HS (37 °C day/30 °C night) and control on the fifth day (**A**). Seed setting rates of OE-1, OE-2, and WT under HS (37 °C day/30 °C night) and control on the fifth day (**B**,**C**). Pollen viability of OE-1, OE-2, and WT under HS and control on the fifth day (**D**). Data shown are means ± SD of three independent experiments. * Significance at *p* < 0.05. ** Significance at *p* < 0.01, as determined by Student’s *t*-test (*n* = 3).

To further determine whether *OsNCED1* is involved in regulating HS tolerance in rice, the function of *OsNCED1* in HS tolerance was investigated by using two Nipponbare transgenic lines with the overexpression of *OsNCED1* cDNA. First, the transcription levels of the *OsNCED1* gene in WT and two overexpression lines were assessed under control and HS treatments. The results showed that OE-1 and OE-2 had significantly higher transcription of *OsNCED1* (Figure 6A). *OsNCED1* was upregulated in WT, OE-1, and OE-2 under HS treatment, and the upregulation was more significant in OE-1 and OE-2 relative to that in WT (Figure 6A). The WT and two transgenic lines showed almost identical seed setting rates under the control conditions. However, under HS treatment, WT, OE1, and OE2 had significantly lower seed setting rates compared with their respective control treatments, and OE-1 and OE-2 exhibited less dramatic decreases and thus higher seed setting rates than WT (Figure 6B,C). The pollen viability under HS showed a generally consistent trend with the seed setting rate (Figure 6D). Compared with the control, WT showed a more significant decrease (by 52.6%) in pollen viability than OE-1 (by 13.91%) and OE-2 (by 15.37%) under HS (Figure 6D). These results suggested that overexpression lines underwent lower degrees of damage under HS than WT, and *OsNCED1* overexpression could improve the HS tolerance of rice at the heading and flowering stage.

### 3.6. Analysis of Membrane Permeability and Antioxidant Enzyme Activity of OsNCED1 Transgenic Rice

**Figure 7:**
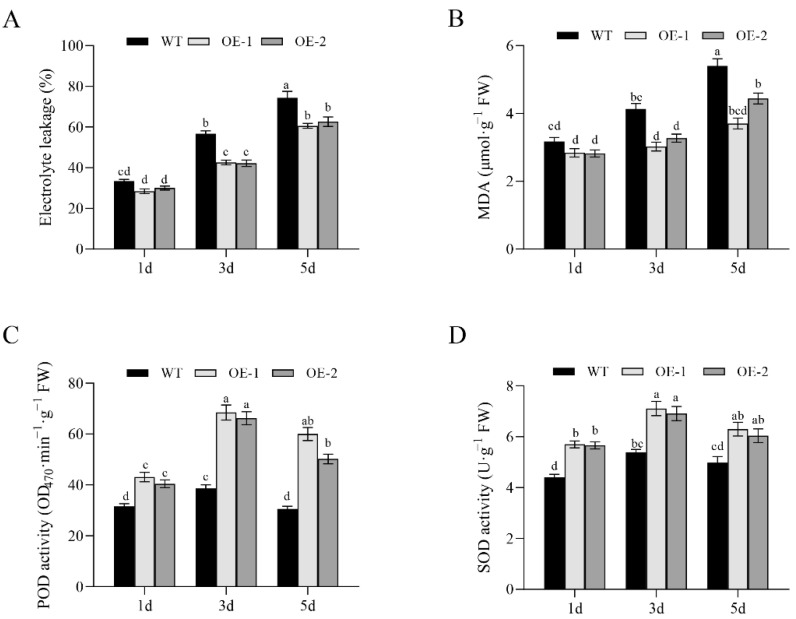
Results from determinations of change (%) of electrolyte leakage (**A**), malondialdehyde content (**B**), peroxidase activity (**C**), and superoxide dismutase activity (**D**) performed in samples collected throughout a five-day HS experiment in plants of two transgenic rice lines OE-1 and OE-2 overexpressing the *OsNECD1* gene, as compared with untransformed plants (WT). Values are mean average ± SD of three replicates; values followed by the same letter were not significantly different at the 0.05 level, according to Duncan’s multiple range test.

To dissect the potential physiological mechanism by which *OsNCED1* enhances HS tolerance, we determined the MDA content, electrolyte leakage, and activities of several antioxidant enzymes in *OsNCED1* overexpression and WT plants under HS. Under HS treatment, both lines showed continuous increases in electrolyte leakage and MDA content in flag leaves with time (Figure 7A,B). However, the electrolyte leakage and MDA content of WT flag leaves under HS were significantly higher than those of lines OE-1 and OE-2 (Figure 7A,B). Therefore, the cell membrane status of these *OsNCED1*-overexpressing lines was less affected by HS that that of WT plants, which would indicate higher heat tolerance.

We next assayed the activities of POD and SOD, two important antioxidant enzymes, in two *OsNCED1* transgenic lines and WT plants under HS (Figure 7C,D). POD and SOD are key enzymes for the scavenging of reactive oxygen free radicals in plants, and their activities are closely related to the antioxidant capacity of plants. Irrespective of the genotype, POD and SOD activities in rice plants increased after 3 days under HS, while they decreased at the end of the experiment treatment (5 days). During the whole experiment, POD and SOD activity levels determined in transgenic lines overexpressing the *OsNECD1* gene were significantly higher than those determined in WT plants. These results could indicate a better reactive oxygen species (ROS) homeostasis maintenance in transgenic plants under HS conditions.

### 3.7. Changes in the Transcription of ABA-Signaling-Related Genes in OsNCED1 Transgenic Rice Plants

**Figure 8:**
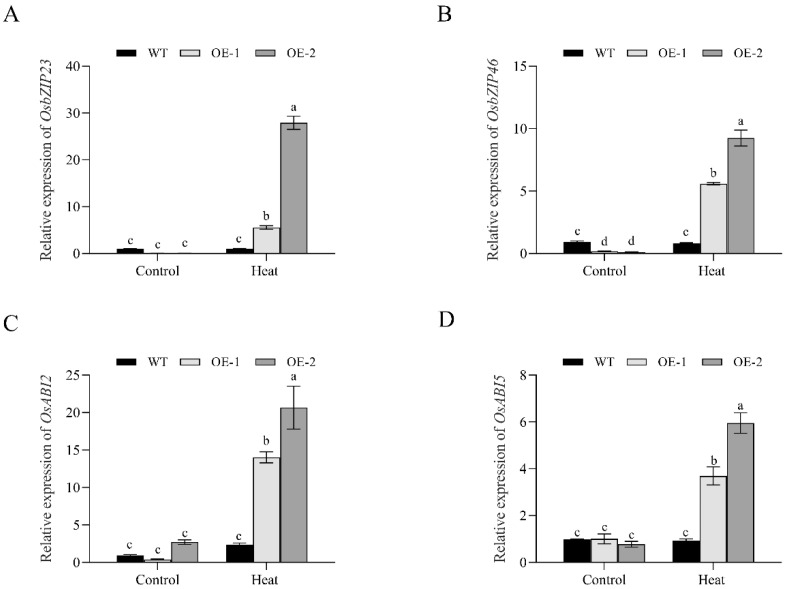
Transcription of ABA signaling-related genes, including *OsbZIP23* (**A**), *OsbZIP46* (**B**), *OsABI2* (**C**) and *OsABI5* (**D**), were analyzed by qRT-PCR under HS in WT and transgenic lines. WT and overexpression lines were exposed to HS (45 °C) for 5 days, and leaf samples were collected for RNA extraction, cDNA synthesis, and qRT-PCR analysis. Data shown are means ± SD of three independent experiments. Statistical analysis was performed by ANOVA test (*p* < 0.05), and significant differences are indicated by different letters.

We compared the transcription levels of several ABA-signaling-related genes between transgenic lines and WT under control or HS conditions by qRT-PCR, including *OsbZIP23*, *OsbZIP46*, *OsABI2*, and *OsABI5*. As shown in Figure 8, *OsNCED1* transgenic lines had significantly higher transcription of these genes than WT plants under HS treatment. In contrast, under control conditions, no significant difference was observed in these genes between WT and transgenic lines, except for *OsbZIP46*. These results suggested that *OsNCED1* overexpression can activate the expression of ABA-signaling-related genes under HS.

## 4. Discussion

Rice is of high sensitivity to HS, particularly at the heading and flowering stages. When the daily average temperature is higher than 32 °C and the daily maximum temperature exceeds 35 °C, HS can decrease the pollen viability to cause a lower seed setting rate and yield, as well as significantly increase the chalky grain rate, affecting starch and cooking quality and reducing the sensory attributes [23,24,25]. Microarray is a powerful tool to be used for analyzing gene expression profiles in plants under exposure to various abiotic stresses [26,27]. In the present study, thousands of DEGs were identified between two genotypes with contrasting response to HS. GO and MapMan analysis demonstrated that the HR genes are mainly involved in cellular process, protein binding, catalysis, and stress response; these findings are consistent with the findings for the HS response of pepper and grape [28,29]. Moreover, the qRT-PCR validation revealed that the qRT-PCR results of the expression of all candidate genes were highly consistent with the microarray data. These annotations can make a valuable resource for the exploration of specific processes, functions, and pathways associated with plant HS tolerance.

TFs play important roles in controlling the expression of relevant genes in response to various stresses. Previously, it has been reported that heat, drought, and virus infection could significantly upregulate the AP2/ERF family genes [30]. Recent studies have demonstrated that water deficit, cold, and salt stress could strongly induce *OsWRKY24* and *OsWRKY28*, while they could greatly suppress the transcription of *OsWRKY82* [31]; and that heat and drought stress upregulated *OsWRKY11* [32]. Research on *NAC* family genes has suggested that the SNAC factor from this family can be utilized to improve the stress tolerance of transgenic plants [33] and that *OsNAC5* can improve the drought stress tolerance of rice by upregulating the stress-inducible genes [34]. Another study of *MYB* family genes revealed that 10 *MYB* genes were induced under the early treatment of HS, while *Os02g0114800* and *Os07g0137000* were induced by the late treatment of HS [35]. In this study, most genes from the *MYB*, bZIP, and *NAC* families were upregulated in HTL252 but downregulated in HSL082 (Supplementary Table S2). HTL252 had much higher expression levels of the C2C2 gene for *Os02g0606200* and *Os01g0263900* than HSL082. In addition, heat shock transcription factors (Hsfs) play an important role in both basal and acquired HS tolerance [36]. In a recent study, HSP70s were found to be upregulated under cold, high salinity, drought, and heavy-metal stresses [37]. In the present study, the HSP70 genes *Os03g0218500* and *Os03g0277300* were significantly upregulated in HTL252, but the HSP70 genes *Os01g0180800*, *Os11g0703900*, *Os12g0244100*, and *Os12g0569700* were downregulated in HSL082. Heat shock proteins (HSPs) are an important class of genes involved in the response of plants to HS, which can be regulated by Hsfs. Some HSPs, such as HSP101, HSP90, HSP60, HSP40, and small heat shock protein (sHSP), which have been identified as molecular chaperones influencing protein quality, were reported to be upregulated under HS [38,39]. HSPs are involved in both plant stress response and normal development; most are significantly upregulated under HS [40]. Our data revealed that most HSPs genes were upregulated in HTL252 but downregulated in HSL082 under HS (Figure 3A and Supplementary Table S3). These results suggest that the upregulated TFs and HSPs may play crucial roles in the response of a tolerant genotype to HS.

ABA is an important plant hormone that regulates plant growth and development and stress resistance. Under drought and salt stress, ABA can induce the expression of genes related to stress resistance and antioxidant activity in plants to improve plant stress tolerance [41,42]. Under HS stress, exogenous ABA could improve the HS tolerance of maize and rice [43,44]. Previous work has demonstrated that the ABA content in rice anthers would increase under HS, indicating that pollen viability/floret sterility may be correlated with the changes in ABA caused by HS [45]. In addition, it has been demonstrated that, under HS, ABA pretreatment could significantly enhance the expression of sHSP17.2, sHSP17.4, and sHSP26 proteins in maize leaves [41]. In the present study, five ABA-related genes were upregulated in HTL252 under HS, but most ABA-related genes were downregulated in HSL082 (Supplementary Table S6). Notably, the *Os02g0704000* (*OsNCED1*) gene was upregulated under HS in both lines, and it exhibited a much higher expression level in HTL252 than in HSL082. These data are in agreement with previous reports, thus indicating the important role of ABA in rice response to HS.

To determine whether the key DEGs identified in transcriptome data are involved in regulating the tolerance to prolonged HS at the heading and flowering stage in rice, we investigated the response of the transgenic plants overexpressing the key DEG *Os02g0704000* (*OsNCED1*) under HS. *OsNCED1* encodes a rate-limiting enzyme in the ABA synthesis pathway, namely the 9-cis-epoxysteroid dioxygenase [46]. Previous studies have shown that the *NCED* gene is highly expressed under stress, regulating the synthesis of endogenous ABA [47] and improving plant stress resistance [48]. The NCED family has 13 members in rice, which have been shown to be involved in the response of plants to abiotic stresses. For example, overexpression of *OsNCED3* and *OsNCED4* in wild-type *Arabidopsis* increased the ABA content and enhanced drought tolerance [49,50]. *OsNCED1* is the housekeeping gene in the *NCED* gene family [51]. Previous studies have demonstrated that the expression of the *OsNCED1* gene is affected by low temperature, drought, and dehydration [52,53,54]. In this study, we uncovered the potential role of *OsNECD1* in rice response to prolonged HS at the heading and flowering stage in rice, since transcript levels of this gene were significantly higher in stress conditions as compared to CT conditions, particularly in the tolerant genotype, for which pollen viability and seed setting rate of overexpression lines under HS were significantly higher than those of WT. These results indicated that *OsNCED1* overexpression could improve the HS tolerance of rice at the heading and flowering stage.

HS can damage the structure and function of cell membranes, disrupting membrane integrity and fluidity, and, in turn, causing various risks to plants [55]. Electrolyte leakage, antioxidant enzyme activity, and MDA content are generally taken to evaluate membrane damage and oxidative damage, as well as to reflect the HS tolerance of plants [56,57]. In this study, *OsNCED1* overexpression lines showed significantly lower electrolyte leakage and MDA content, while we noted higher SOD and POD activities in flag leaves than the WT at days 1, 3, and 5 after HS treatment. These results suggest that *OsNCED1* may enhance antioxidant capacity by activating these antioxidant enzymes to reduce membrane lipid peroxidation and thus improve HS tolerance. Consistently, another study reported that *OsANN1* overexpression could improve the HS tolerance of rice by increasing CAT and SOD activities [58]. Similarly, Sailaja et al. [59] demonstrated that the heat-tolerant rice variety Nagina22 has higher antioxidant enzyme activities and lower ROS and MDA contents than the heat-susceptible rice variety Vandana. Moreover, our data demonstrated that *OsNCED1* overexpression could significantly increase pollen viability and seed setting rate under HS. For rice treated with 38 °C day/30 °C night at the meiosis stage, the ROS content in the anther was more than three folds that under normal temperature treatment, and this eventually led to a significant reduction of pollen viability and floret fertility [60]. Li et al. [61] found that excessive ROS accumulation leads to severe oxidative stress on microspores during pollen development, and this, in turn, triggers programmed cell death, resulting in microspore abortion. Therefore, an appropriate level of ROS concentration is essential for pollen activity and seed setting in rice.

There has been increasing evidence suggesting that the regulation of ROS by ABA signaling plays an active role in the HS tolerance of plants [62,63,64,65]. In this study, the transcription levels of ABA-signaling-related genes, including *OsbZIP23*, *OsbZIP46*, *OsABI2*, and *OsABI5*, were significantly elevated in *OsNCED1* overexpression lines under HS treatment. Numerous studies have demonstrated that these key genes are involved in the ABA signaling pathway to regulate the tolerance of plants to multiple stresses [66,67]. For instance, bZIP23 can directly interact with the promoter of the seed-specific antioxidant *PER1A* to mediate the detoxification pathway and improve rice seed viability [68]. In *Arabidopsis*, ABI5 affects ROS homeostasis by activating CAT1 expression and influencing peroxidase activity [69]. Miao et al. [70] showed that *Arabidopsis GLUTATHIONE PEROXIDASE3* (ATGPX3) interacts with ABI2 and mediates H_2_O_2_ homeostasis. In addition, the overexpression of *SgNCED1* in tobacco could increase the antioxidant enzyme activity and enhance its drought and salt tolerance [71]. In general, our results suggest that *OsNCED1* may enhance the activity of antioxidant enzymes by regulating the transcription of ABA-signaling-related genes, thereby improving the HS tolerance of rice. This study preliminarily reveals the role of the *OsNCED1* gene in rice HS tolerance and the underlying mechanism. The biological function and specific signaling pathway of *OsNCED1* need to be further studied.

## 5. Conclusions

HS is an important adverse environmental factor that negatively affects the yield and quality of rice. The genome-wide analysis in this study showed that the genes in response to prolonged HS during the heading and flowering of rice are mainly enriched in cellular response, regulation, and metabolism. In addition, genes for HSPs, TFs, UPS, and phytohormones may play important roles in the response of heat-tolerant variety (HTL252) to HS. The overexpression of *OsNCED1* could increase pollen viability and seed setting rate under HS, suggesting that *OsNCED1* positively regulates HS tolerance in rice at the heading and flowering stage, and this can be at least partly attributed to better maintenance of ROS homeostasis. This study provides important implications for the genetic improvement of HS tolerance in plants.

## Data Availability

The data presented in this study are available upon request from the corresponding author.

## Supplementary Materials

The following are available online at https://www.mdpi.com/article/10.3390/plants11081062/s1. Table S1: Primers list. Table S2: Transcription factors. Table S3: Heat shock proteins. Table S4: Development-related genes. Table S5: Ubiquitin-mediated proteolysis system. Table S6: Hormone-signaling-related genes.
